# METTL14 suppresses pyroptosis and diabetic cardiomyopathy by downregulating TINCR lncRNA

**DOI:** 10.1038/s41419-021-04484-z

**Published:** 2022-01-10

**Authors:** Liping Meng, Hui Lin, Xingxiao Huang, Jingfan Weng, Fang Peng, Shengjie Wu

**Affiliations:** 1 Department of Cardiology, Shaoxing People’s Hospital(Shaoxing Hospital, Zhejiang University School of Medicine), Shaoxing, 312000 Zhejiang China; 2 Department of Cardiology, the First Affiliated Hospital of Wenzhou Medical University, Wenzhou, China

**Keywords:** Cardiomyopathies, Endocrine system and metabolic diseases

## Abstract

N6-methyladenosine (m6A) is one of the most important epigenetic regulation of RNAs, such as lncRNAs. However, the underlying regulatory mechanism of m6A in diabetic cardiomyopathy (DCM) is very limited. In this study, we sought to define the role of METTL14-mediated m6A modification in pyroptosis and DCM progression. DCM rat model was established and qRT-PCR, western blot, and immunohistochemistry (IHC) were used to detect the expression of METTL14 and TINCR. Gain-and-loss functional experiments were performed to define the role of METTL14-TINCR-NLRP3 axis in pyroptosis and DCM. RNA pulldown and RNA immunoprecipitation (RIP) assays were carried out to verify the underlying interaction. Our results showed that pyroptosis was tightly involved in DCM progression. METTL14 was downregulated in cardiomyocytes and hear tissues of DCM rat tissues. Functionally, METTL14 suppressed pyroptosis and DCM via downregulating lncRNA TINCR, which further decreased the expression of key pyroptosis-related protein, NLRP3. Mechanistically, METTL14 increased m6A methylation level of TINCR gene, resulting in its downregulation. Moreover, the m6A reader protein YTHDF2 was essential for m6A methylation and mediated the degradation of TINCR. Finally, TINCR positively regulated NLRP3 by increasing its mRNA stability. To conclude, our work revealed the novel role of METTL14-mediated m6A methylation and lncRNA regulation in pyroptosis and DCM, which could help extend our understanding the epigenetic regulation of pyroptosis in DCM progression.

## Background

Diabetic cardiomyopathy (DCM), a major cardiovascular complication of diabetes, is characterized by myocardial fibrosis, ventricular remodeling, and cardiac dysfunction [1]. DCM is closely associated with the occurrence of heart failure, making it the majority cause of death among patients with diabetes [2]. DCM influences heart healthy through various mechanisms, including changes in metabolism, abnormal subcellular composition, and damage of microvascular [3]. However, the detailed mechanism of DCM is not well known and remains elusive. Revealing the key genes involved in DCM and identifying the potential regulatory mechanism will provide therapeutic targets used for overcoming DCM.

Pyroptosis is characterized by rapid plasma membrane rupture, with the consequent release of intracellular contents and pro-inflammatory mediators, such as caspase-1 [4]. The main signaling pathway involved in pyroptosis is mediated by caspase-1 activation, resulting in the maturation process of IL-1β, IL-18, and gasdermin D (GSDMD) [5]. Previous studies widely reported the essential role of pyroptosis in cardiomyopathy, especially in DCM [6]. Xie et al. demonstrated that NLRP3 inflammasome mediated the chemerin/CMLR1-induced inflammation and pyroptosis and contribute to DCM [7]. Another research by Cao et al. revealed that high glucose-induced cardiotoxicity by inhibiting NLRP3 inflammasome activation and pyroptosis [8].

As the most abundant chemical modification of eukaryotic messenger RNA (mRNA), N6-methyladenosine (m6A) is known to influence various fundamental bioprocesses by regulating target gene expression [9]. m6A regulatory proteins are composed of the “erasers” FTO and ALKBH5, the “readers” YTHDFs and IGF2BPs, and the “writers” METTL3, METTL14 and WTAP [10]. Alterations in the m6A level mediate cell apoptosis, proliferation, self-renewal, and development [11]. However, the potential role of m6A methylation in DCM progression remains unknown.

Methyltransferase-like 14 (METTL14), a well-known m6A writer protein, widely participated in the progression of major diseases, such as cardiovascular pathogenesis [12]. However, whether METTL14 regulates DCM and the underlying mechanism are under investigation. In this study, we revealed that METTL14 was downregulated in myocardium tissues of DCM rat, and high glucose suppressed the METTL14 expression level. Mechanistically, the METTL14-mediated activity of m6A modification of TINCR suppressed pyroptosis of cardiomyocytes and DCM in an NLRP3-dependent manner.

## Materials and methods

### Cell lines and chemical reagent

As we previously described [13], neonatal rat ventricular myocytes (NRVMs) were obtained from isolated heart tissues of young Wistar rats. Briefly, the dissected hearts minced in HEPES-buffered nic acid saline solution. Non-myocyte contaminants were removed by two rounds of pre-plating for 1.5 h on 100-mm plastic cell culture dishes under a culture condition of 37 °C and 5% CO_2_. Then, the cardiomyocytes were seeded into different culture dishes with medium containing serum. After incubation for 24 hours, medium without serum was used to replace the serum-containing medium.

The normal H9c2 cardiomyocyte cell line was purchased from American Type Culture Collection (ATCC). Cells were cultured with 1640 medium containing 10% fetal bovine serum in a humidified atmosphere of 5% CO_2_ at 37 °C. Actinomycin D was purchased from Sigma-Aldrich (St. Louis, MO, USA) and used at the concentration of 5 μg/ml. MCC950 sodium (CP-456773, CRID3 sodium salt), a NLRP3 inhibitor, was used at the concentration of 7.0 nM in cell culture and 12 mg/kg in animal treatment (Selleck, Shanghai, China). For osmotic stress, hyperosmotic medium was made by adding various concentrations of D-sorbitol (Sigma-Aldrich) to regular 1640 medium for different durations. Standard 1640 culture condition was used as isosmotic media.

### Establishment and treatment of diabetic animal models

A total of 120 Male Sprague-Dawley rats weighing 200-250 g (95-110 days old) were purchased from Model Animal Research Center of Nanjing University were randomly divided into respective treatment groups (*n* = 8 per group). The diabetic model was constructed by a single intraperitoneal injection of streptozotocin (65 mg/kg), which imitates a model of type 1 diabetes. The fasting blood glucose was measured one week after injection. Only rats with glucose levels higher than 16.7 mmol/L were defined as diabetic. Cardiac function was investigated seven days following the last treatment, and the heart tissues were then isolated for expression analyses. The lentivirus vector used for silencing or overexpressing specific genes were dissolved in 50 μL saline at the concentration of 1 × 10^9^ TU with one dose after the animal model was established. NLRP3 inhibitor MCC950 (10 mg/kg) was intraperitoneally injected 30 min before streptozotocin treatment. The experimental protocol was approved by IACUC of Shaoxing Hospital of Zhejiang University (Shaoxing city, China).

### RNA extraction and quantitative RT-PCR

Total RNA was isolated by TRIzol reagent (Invitrogen, Carlsbad, CA). A total of 1 mg of extracted RNA was used to perform reverse transcription using Superscript III transcriptase (Invitrogen). Quantitative real-time PCR (qRT-PCR) was performed using SYBR Green real-time PCR analysis using Bio-Rad CFX96 system (Bio-Rad, Cambridge, MA) with the specific primers. Expression of the PCR data were shown as 2^−ΔΔct^, and normalized to the internal control level (GAPDH).

### Echocardiography

Rats from respective treatment groups were fixed on operating table and were performed under sodium pentobarbital anesthesia (75 mg/kg), and all efforts were made to minimize suffering. Echocardiography was performed using a Vivid 7 Dimension (GE Healthcare, Munich, Germany) echocardiograph equipped with a 14-MHz transducer. Parasternal short-axis views were used for M-mode analysis. Enddiastolic and endsystolic left ventricular inner diameters (LVIDd, LVIDs) were measured, and fractional shortening (FS) and left ventricular ejection fraction (LVEF) were calculated using the following equations: FS = (LVIDd-LVIDs)/LVID, LVEF = (LVIDd^3^ − LVIDs^3^)/LVIDd^3^.

### Transmission electron microscopy

Hearts were extracted, cut finely into small (~1 mm^3^) blocks, and fixed overnight in 4% glutaraldehyde in 100 mM phosphate buffer, followed by post-fixation in 2% osmium tetroxide in 100 mM phosphate buffer. Specimens then underwent en bloc treatment with uranyl acetate, dehydration in ethanol, and transferred to propylene oxide, prior to embedding. Ultra-thin sections (50–70 nm) were cut and stained with uranyl acetate and lead citrate, and examined in a JEOL 1200EX electron microscope.

### TUNEL assay

Cells were firstly fixed with 4% paraformaldehyde for 30 min followed by staining with one-step TUNEL kit as per the instructions of the manufacture (Beyotime, Shanghai, China). The positive stained parts were visualized and calculated via a fluorescence microscopy (Axio Observer A1, ZEISS, Germany).

### IHC analysis

Heart myocardial tissues were isolated and fixed with 4% Paraformaldehyde followed with incubation with primary antibodies against respective proteins: NRLP3 (1:100, cat. no. ab214185, Abcam, Cambridge, MA), METTL14 (1:100, ab223090, Abcam), cleaved caspase-1 (1:100, ab1872, Abcam) and GSDMD-N (1:100, bs-14287R, Bioss, Beijing, China), to detect their expression levels. Images were visualized using a ZEISS Axio Observer A1 (Oberkochen, Germany) microscope system and processed with ZEISS software.

### Masson staining

After isolation of rat heart from respective treatment groups, the tissues were fixed with 4% paraformaldehyde followed by embedding with paraffin and sectioning. Masson staining process was done by using Masson’s Trichrome Stain Kit as per the guidelines of manufacture (cat.no.G1340, Solarbio, Beijing, China). The sections were treated sequentially with hematoxylin and ferric oxide, acid fuchsin, phosphomolybdic acid, and acetic acid. The quantification was performed with Image J following mounting of the sections with neutral gum.

### RIP and RNA pulldown assay

The RNA immunoprecipitation (RIP) was performed using the EZ-Magna RIP kit (Millipore, Burlington, MA, USA) according to the manufacturer’s instructions. Briefly, 10^7^ cells were lysed with RIP lysis buffer using one freeze-thaw cycle. Cell extracts were coimmunoprecipitated with anti-m6A (ab208577, Abcam) and NLRP3 (ab263899, Abcam) antibodies and the retrieved RNA was subjected to qRT-PCR analysis.

The RNA pulldown assay was performed using a Magnetic RNA-Protein Pull-down Kit (Thermo Scientific) according to the manufacturer’s instructions. The 3ʹ-end Biotin-TEG modified-DNA probes against TINCR were synthesized by Sangon (Shanghai, China). The cell lysates were hybridized with a mixture of biotinylated DNA probes for 4 h at 37 °C. The binding complexes were then recovered using streptavidin-conjugated magnetic beads. Finally, protein was eluted and purified from the beads for western blot analyses.

### Western blots

Protein extraction was performed using RIPA lysis buffer (Pierce, IL, USA) containing protease inhibitor (Roche, CA, USA). Protein extracts were subjected to 10% SDS-polyacrylamide gel electrophoresis followed by electro-transfer to polyvinylidene difluoride membrane. After 1 h of pre-membrane blocking with 5% BSA, the proteins were incubated with respective primary antibodies at 4 °C overnight followed by secondary antibodies incubation at room temperature for 1 h. The detection of proteins was carried out using ECL reagent. Primary antibodies used in this study include: NRLP3 (1:1000, cat. no. ab214185, Abcam), METTL14 (1:1000, ab223090, Abcam), cleaved caspase-1 (1:1000, ab1872, Abcam), IL-1β (1:1000, ab2105, Abcam) and IL-18 (1:1000, ab18672, Abcam), GSDMD-N (1:1000, bs-14287R, Bioss, Beijing, China) and GAPDH (Invitrogen, cat. no. PA1-987).

### Statistical analysis

All experiments were performed in triplicate. Statistics were presented as mean ± SD. Comparison between two groups was analyzed using the Student’s *t*-test. Fisher exact testing was performed to evaluate the difference of proportions between different groups. Statistical analyses were performed using GraphPad Prism (v8.0.1, GraphPad Software Inc., San Diego, CA, USA). *P* < 0.05 was considered to indicate a statistically significant difference.

## Results

### Pyroptosis is involved in DCM through NLRP3-caspase-1 pathway in vitro

**Fig. 1:**
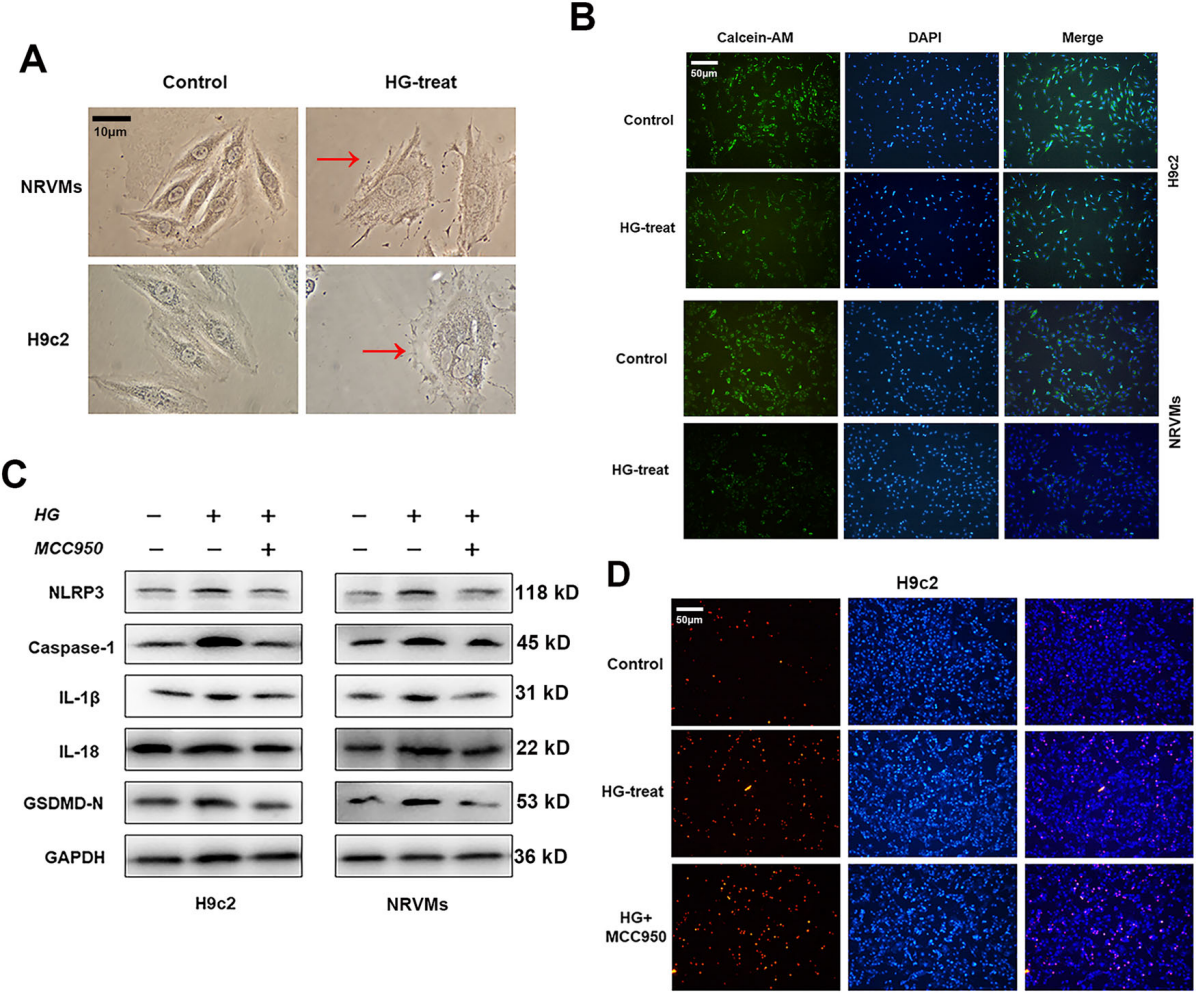
HG treatment induces pyroptosis through targeting NLRP3. **A** Morphologic changes of cells cultured with HG were consistent with pyroptosis, including swelling changes and rupture of cell membrane. **B** Calcein-AM (dyed with green) staining of HG-treated and controlled cardiomyocytes were shown. **C** Western blots were performed to detect the expression level of pyroptosis-related proteins in cardiomyocytes treated with HG and/or NLRP3 inhibitor MCC950 (5 μg/ml) for 24 h. **D** TUNEL staining was used for evaluating the apoptosis level of H9c2 cells treated with HG and(or) MCC950 (10 mg/kg).

We treated neonatal rat ventricular myocytes (NRVMs) and cardiomyocyte cell line H9c2 with glucose at the concentration of 5.5 mmol/L (normal control) or 50 mmol/L (high glucose, HG) to imitate the hyperglycemic condition. HG status was generated with the concentration of 50 mmol/L since another reported concentration used in HG was not enough for activating pyroptosis, such as 25 mmol/L (Supplementary Fig. S1A). As shown in Fig. 1A, due to the HG treatment, H9c2 cells exhibited characteristic morphological changes, such as swelling changes and rupture of the cell membrane, suggesting that pyroptosis was directly involved in DCM process. Then we performed Calcein-AM staining analysis and found that HG treatment caused an increased damage of cell membrane when compared with control treatment (Fig. 1B and Supplementary Fig. S1B). Moreover, the protein expression of pyroptosis markers, including NLRP3, cleaved caspase-1, and GSDMD-N, were significantly upregulated in HG-treated cardiomyocytes. Importantly, treatment with MCC950, a well-known NLRP3 inhibitor, suppressed pyroptosis-related proteins and dramatically reversed pyroptosis of cells treated with HG (Fig. 1C and Supplementary Fig. S1C). We also evaluated the effect of HG treatment on cell apoptosis by performing TUNEL staining. As shown, apoptosis was activated upon HG treatment, however, this activation was not restored by MCC950 treatment in H9c2 cells (Fig. 1D and Supplementary Fig. S1D). For in vivo data, IHC showed a decreased expression of NLRP3 upon MCC950 treatment (Supplementary Fig. S1E). However, echocardiography and ELISA showed that MCC950 showed no effect on cardiac function and inflammation (Supplementary Fig. S1F, G). Finally, we treated H9c2 cells with D-sorbitol at different concentrations, representing hyperosmotic pressures, to verify the effects of hyperosmotic condition on pyroptosis. As shown, pyroptosis-related proteins were not activated under sole hyperosmotic conditions (Supplementary Fig. S1H), which excluded the involvement of hyperosmotic pressure during HG treatment-induced pyroptosis. These data showed that HG treatment resulted in pyroptosis of cardiomyocytes in an NLRP3-dependent manner.

### Pyroptosis was activated in cardiomyocyte of DCM rats

**Fig. 2:**
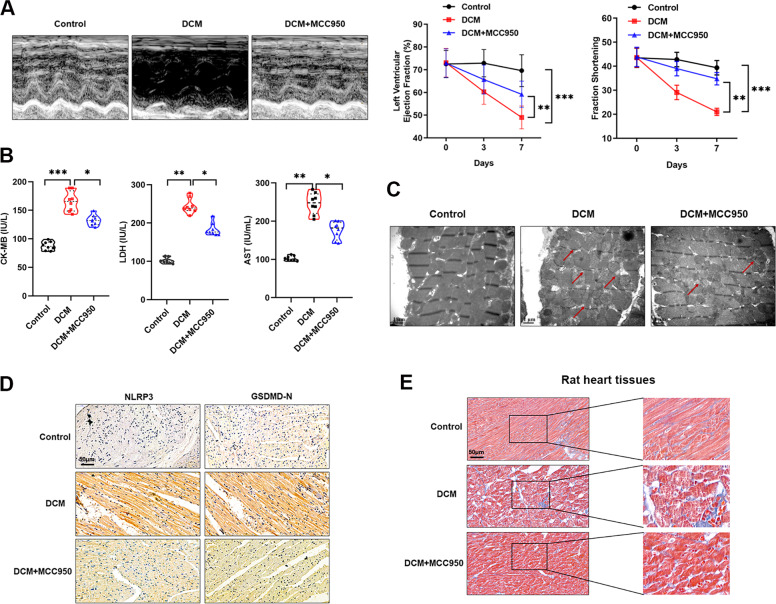
Pyroptosis was involved in DCM in vivo. **A** Morphological changes (left panel) and quantitative analysis of LVEF and FS (right panel) in rat hearts were detected by echocardiography, **P < 0.01, ***P < 0.001. **B** Serum expression of myocardial enzymes CKMB, LDH and AST were quantified in rats of respective groups, *P < 0.05, **P < 0.01, ***P < 0.001. **C** A representative section of the left ventricle from respective groups of rats showing sporadic mitochondrial, damaged disconnected myofibers, and thinner myofibers. **D** IHC analysis was performed to detect the expression of NLRP3 and GSDMD-N in hearts of DCM rat after treated with MCC950. **E** Masson staining was done in heart tissues of DCM rats treated with MCC950.

To further verify the essential role of pyroptosis in HG-induced toxicity of cardiomyocytes, we constructed DCM model using Wistar rats by single intraperitoneal injection of streptozotocin, an animal model that mimics type 1 diabetes. Echocardiography was performed to evaluate the cardiac function of DCM rats. Our data revealed a reduced LVEF and FS in rats with DCM in contrast to normal rats. The LVEF level of DCM rats was 48%, which is significantly lower than that in normal rats (68%). Moreover, the average FS of rats with DCM was also dramatically decreased compared to control rats (21% vs. 37%) (Fig. 2A). Interestingly, when NLRP3 was silenced with MCC950 in DCM rats, the damaged cardiac function caused by DCM was dramatically reversed (Fig. 2A). By detecting myocardial enzyme markers, such as AST, LDH, and CK-MB, we identified an elevated expression in DCM rats, however, MCC950 treatment partly restored this effect. (Fig. 2B). In addition, electron microscopy imaging of cardiomyocyte ultrastructure showed that DCM rats showed serious cardiomyocyte damage compared to control hearts, including increased inter-mitochondrial distance, disconnected cardiac myofibers, and thinner myofibers, and this damage could be partially rescued by MCC950 (Fig. 2C). Finally, the expressions of pyroptosis proteins, NLRP3 and GSDMD-N, were dramatically increased in rats of DCM compared, meanwhile, this influence was significantly relieved by MCC950 (Fig. 2D). Since pyroptosis is accompanied with rupture of cardiomyocyte membrane, causing the damage and disruption of collagens, thus the collagen status could reflect the process of pyroptosis. Masson staining showed that collagen was damaged in DCM rats and further reversed by MCC950 treatment (Fig. 2E). Taken together, we demonstrated that DCM was closely associated with cardiomyocytes pyroptosis in an NLRP3-dependent manner.

### METTL14 is downregulated in DCM

**Fig. 3:**
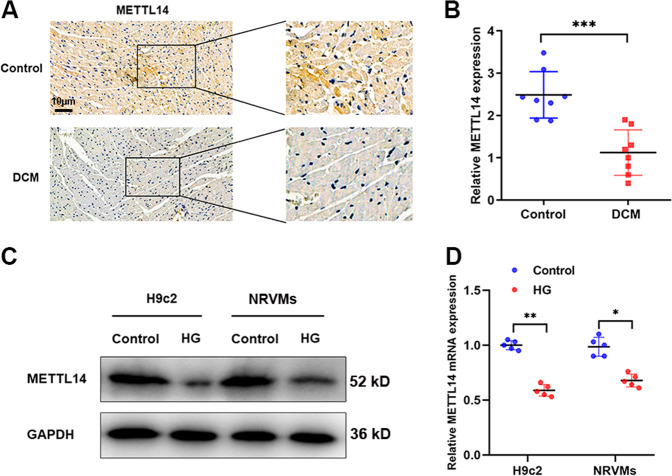
METTL14 was downregulated in DCM rats. **A** METTL14 protein expression was detected in heart tissues of DCM and control rats. **B** Serum circulating METTL14 mRNA was determined in rats of respective groups, ***P < 0.001. **C**, **D** METTL14 protein (**C**) and mRNA (**D**) levels were detected in cardiomyocytes treated with HG, *P < 0.05, **P < 0.01.

To find whether METTL family was involved in pyroptosis in DCM models, we detected the expression level of METTL14 and METTL3 in DCM rats. As shown in Fig. 3A, B, METTL14 was significantly downregulated in heart tissue and serum samples of DCM rats compared to those of controlled rats. Moreover, a decreased METTL14 level was identified in NRVMs and H9c2 cells treated with HG when compared with control cells (Fig. 3C, D). Specially, METTL14 level was not dysregulated under hyperosmotic pressures with D-sorbitol treatment (Supplementary Fig. S2A). On the other hand, METTL3 expression was not altered according to our in vitro and in *vivo* data (Supplementary Fig. S2B, C). These indicate that METTL14 may be a key molecular regulator during DCM initiation and progression.

### METTL14 suppresses DCM via modulating pyroptosis

**Fig. 4:**
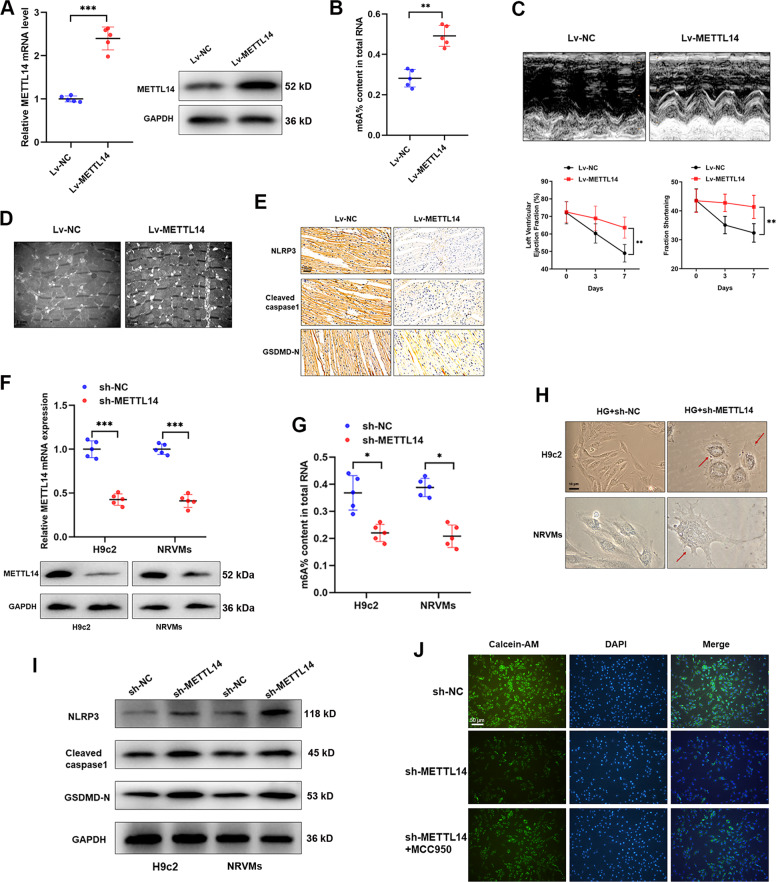
METTL14 suppresses DCM via modulating pyroptosis. **A** Validation of METTL14 overexpression in DCM rats by western blot and qRT-PCR, ***P < 0.001. **B** The overall m6A content was increased by upregulation of METTL14, **P < 0.01. **C** Morphological changes (upper panel) and quantitative analysis of LVEF and FS (lower panel) in rat hearts were detected by echocardiography after injection of METTL14 overexpression vector, **P < 0.01. **D** Electron microscopy imaging of cardiomyocyte ultrastructure showed that rats overexpressed with METTL14 showed decreased cardiomyocyte damage compared to control group. **E** IHC analysis of pyroptosis-related proteins in DCM rats overexpressed METTL14. **F** Confirmation of silence of METTL14 in cardiomyocytes at both transcript and protein levels, ***P < 0.001. **G** knockdown of METTL14 caused significantly downregulated m6A modification level, *P < 0.05. **H** Morphologic changes of HG-treated cardiomyocytes upon knockdown of METTL14. **I** Western blot experiment was carried out to reveal the expression changes of pyroptosis-related proteins in cardiomyocytes transfected with METTL14 silencing vectors. **J** Calcein-AM staining showed that deletion of METTL14 increased membrane rupture of cardiomyocytes, however, this effect was reversed by treatment with MCC950.

To clarify the role of m6A modification in pyroptosis and DCM progression, we evaluated the effect of METTL14 in DCM progression. As expected, injection of Lv-METTL14 into DCM rats significantly increased m6A level in DCM rats (Fig. 4A, B). Echocardiography suggested that overexpression of METTL14 increased LVEF, FS (Fig. 4C). Meanwhile, electron microscopy revealed a relieved injury in cardiomyocyte (Fig. 4D). Moreover, enhanced METTL14 inhibited pyroptosis level in myocardial tissues, including downregulation of NLRP3, caspase-1, and GSDMD-N (Fig. 4E), suggesting that METTL14-mediated m6A modification may suppress DCM through modulating pyroptosis. To confirm this hypothesis in vitro, we silenced METTL14 and m6A modification level in H9c2 and NRVMs cells (Fig. 4F, G). Reversed morphologic changes of pyroptosis were observed upon the silence of METTL14 (Fig. 4H). Western blotting showed that sh-METTL14 caused upregulated NLRP3, cleaved caspase-1, and GSDMD-N (Fig. 4I). Consistently, Calcein-AM staining revealed an increased membrane damage in H9c2 cells silenced with METTL14 compared to controls, however, this effect was abrogated by MCC950 (Fig. 4J). Collectively, we proved that METTL14-mediated m6A modification plays essential roles in DCM via regulating cardiomyocyte pyroptosis.

### METTL14 suppresses pyroptosis via targeting TINCR lncRNA

**Fig. 5:**
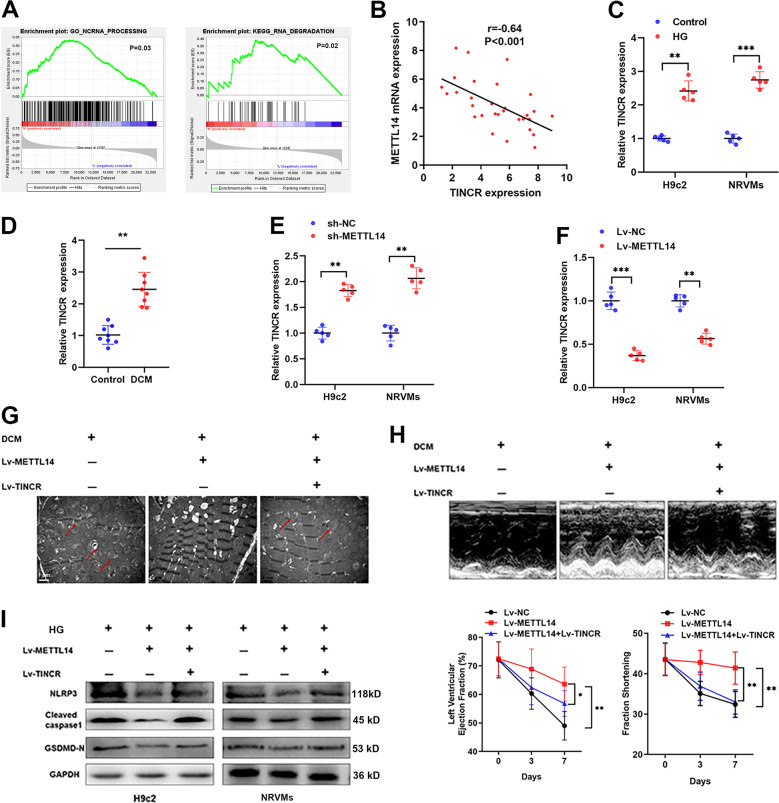
METTL14 suppresses pyroptosis via targeting TINCR lncRNA. **A** GSEA analysis revealed a positive correlation between METTL14 and ncRNA processing and degradation. **B** A significant negative association was identified between METTL14 mRNA and TINCR expression. **C** HG treatment of cardiomyocytes significantly increased TINCR expression, **P < 0.01, ***P < 0.001. **D**. TINCR was significantly upregulated in DCM rats in contrast to controlled rats, **P < 0.01. **E**, **F** qRT-PCR showed that silence of METTL14 (**E**) upregulated, while overexpression of METTL14 (**F**) suppressed TINCR expression in cardiomyocytes, **P < 0.01. **G**, **H** Cardiac function analysis (**G**), electron microscopy imaging (**H**) showed that injection of METTL14 dramatically reversed the myocardial damage caused by DCM, however, co-expression of TINCR abrogated the METTL14-mediated influence, *P < 0.05, **P < 0.01. **I** The expressions of pyroptosis-related proteins was detected in H9c2 and NRVMs cells upon indicated treatments.

It is reported that m6A process was closely associated with lncRNA processing [14]. By performing GSEA analysis, we revealed that METTL14 was involved in the process and degradation of ncRNAs (Fig. 5A). Based on our previous observation of lncRNA TINCR in pyroptosis, we supposed that METTL14 may regulate DCM through TINCR-mediated pyroptosis. To prove this assumption, we detected TINCR expression and found that TINCR was negatively correlated with METTL14 in patients with DCM (Fig. 5B). In addition, TINCR was upregulated in HG-treated cells and DCM rats (Fig. 5C, D). Next, we silenced METTL14 in NRVMs and H9c2 cells, and found that TINCR was upregulated accordingly (Fig. 5E), while overexpression of METTL14 leads to a decreased expression of TINCR (Fig. 5F).

Then, we performed gain-or-loss functional assays by injection of TINCR lentiviral vector into METTL14-overexpressing DCM rats. Intriguingly, overexpression of TINCR reversed the METTL14-induced effects on LVEF, FS, and cardiomyocyte damage in DCM rats (Fig. 5G, H). In addition, enhanced TINCR abrogated METTL14-caused suppression of pyroptosis in H9c2 and NRVMs (Fig. 5I). Meanwhile, suppression of TINCR rescued pyroptosis caused by METTL14 silencing in normal rats as evidenced by western blots, electron microscopy imaging, and echocardiography analyses (Supplementary Fig. S3A–C). Collectively, we proved that lncRNA TINCR was, at least partly, responsible for METTL14-induced suppression of pyroptosis and DCM.

### METTL14-dependent m6A methylation downregulated expression of TINCR

**Fig. 6:**
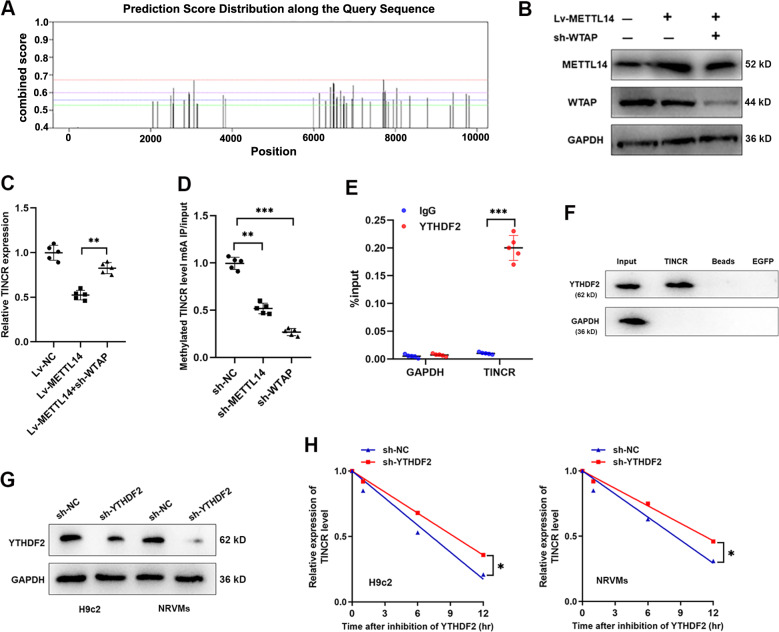
TINCR was downregulated due to the METTL14-mediated m6A methylation. **A** Predicted binding sites of m6A modification at TINCR sequence according to the online SRAMP database (http://www.cuilab.cn/sramp). **B** METTL14-overexpression virus and sh-WTAP vector was injected into DCM rats. Protein band intensity of METTL14 and WTAP were quantified by western blot assay. **C** TINCR expression was determined in rats injected with Lv-METTL14 and(or) sh-WATP, **P < 0.01. **D** Methylated TINCR level was detected via RIP assay, and a significant downregulation by silence of WTAP and METTL14 was observed, **P < 0.01, ***P < 0.001. **E** RIP assay using YTHDF2 antibody revealed a significant association with TINCR, ***P < 0.001. **F** RNA pulldown experiment showed that YTHDF2 was enriched by TINCR probe. **G** Western blot validation of YTHDF2 protein following the transfection of sh-YTHDF2 vector. **H** Two cardiomyocyte cell lines were treated with AtcD, then existing TINCR was detected. Silence of YTHDF2 significantly increased degradation of TINCR, *P < 0.05.

Take a step further, we sought to find whether it is the METTL14-mediated m6A modification that downregulated TINCR expression. By analyzing the potential m6A binding sites with online SRAMP database (http://www.cuilab.cn/sramp), we verified 45 m6A residues located across TINCR sequence (Fig. 6A), among which 11 were identified as *high/very high confidence* (Table 1). It is well demonstrated that METTL14 acted as an interactor with WTAP, binding to the methyltransferase to form a complex which mediates m6A methylation on RNAs. Here, sh-WATP vector was injected into METTL14-overexpressed DCM rats (Fig. 6B). Suppressed TINCR caused by METTL14 was abrogated by silence of WATP (Fig. 6C). Then, we performed RIP assay using m6A antibody, and found a significantly decreased methylated TINCR (site 7709) bounded by m6A upon METTL14 deletion or WTAP deletion (Fig. 6D). The above results strongly suggest that METTL14-dependent m6A modification of TINCR results in its downregulation.

**Table 1:** The predicted 45 m6A residues located across TINCR sequence according to online SRAMP database (http://www.cuilab.cn/sramp).

TaRGET #	Position	Sequence context	Score (binary)	Score (knn)	Score (spectrum)	Score (combined)	Decision
1	214	CCGCUGACCGUGCGGCCGCGGGACACGCUCAGCGACCUGCGCGCC	0.603	0.76	0.573	0.599	m6A site (Moderate confidence)
2	2054	GAAGGGCGCUGGGGCCACGGGAACAGCUCACUGCCGGCCUGGGGC	0.461	0.702	0.653	0.55	m6A site (Low confidence)
3	2169	ACCACCUCCUUGGAAGCAUUGAACAGGAGAGGGACGGGGGCCAUG	0.507	0.599	0.603	0.55	m6A site (Low confidence)
4	2494	UGGACGACCCUCAGACGUCCAGACUGUAAUUAUGCAGUACAGAGU	0.627	0.58	0.525	0.584	m6A site (Moderate confidence)
5	2548	UUGUGUGCCUACUGUGUGCCAGACUCCAUGCUGGACACUGAAAUU	0.49	0.473	0.61	0.537	m6A site (Low confidence)
6	2560	UGUGUGCCAGACUCCAUGCUGGACACUGAAAUUCAGCAGUGACCC	0.635	0.443	0.636	0.626	m6A site (High confidence)
7	2820	UGUGCAAAGGCCCUGAGGCAGGACUGCACCUGGCAUGUUGGAGGA	0.717	0.691	0.325	0.559	m6A site (Moderate confidence)
8	2936	CAGUUCAUGCAGGGCCUUGCGGACUGCAGGGAGGACUUGGGCCUU	0.702	0.727	0.451	0.603	m6A site (High confidence)
9	2948	GGCCUUGCGGACUGCAGGGAGGACUUGGGCCUUGACUCCGAGGGA	0.697	0.73	0.425	0.59	m6A site (Moderate confidence)
10	3060	CUGGCUGCAGCGGGGAGAACAGACUGUGAUGGGGGGAGGGCUGGA	0.752	0.747	0.543	0.668	m6A site (High confidence)
11	3131	UGAGCCAUGCUGGAGGCAGAGAACAGAAGCCUUCAGAGGAGACAG	0.432	0.573	0.677	0.537	m6A site (Low confidence)
12	3150	AGAACAGAAGCCUUCAGAGGAGACAGUUUUGGCUGGGCGCAAUGG	0.474	0.423	0.637	0.536	m6A site (Low confidence)
13	3778	AGAAAUGAGAAAAACACUUGGGACCUGUCAGCGCAGACGGUACCC	0.588	0.629	0.577	0.586	m6A site (Moderate confidence)
14	3833	GAAGCUGCGUGGGCUGCUCGGGACAAACCUGCCAGGCCCUCUCUC	0.6	0.487	0.532	0.567	m6A site (Moderate confidence)
15	5987	UAGUAGUUGAGAAAGAGAAAGGACCUUAGAGAUUGUGGAGACCAU	0.623	0.711	0.483	0.572	m6A site (Moderate confidence)
16	6135	UUGGUGUCUCAUUUCUUUUUGGACUAACACGUGGACCAGAGCCGU	0.661	0.662	0.503	0.598	m6A site (Moderate confidence)
17	6288	CUCUUUGCCUGCAAUUCCUGUGACUGCCAUCAGGUGGCAGAAAAC	0.544	0.474	0.575	0.553	m6A site (Low confidence)
18	6405	UCCAGCCUGGCGACAGAGCAGGACUCUGUCUCAAAAAAAAGAAAG	0.669	0.229	0.63	0.631	m6A site (High confidence)
19	6480	CCAUCCCUGUUCCUCUUAAAGGACACAAGGGACAUCCACAGGAGG	0.65	0.67	0.664	0.656	m6A site (High confidence)
20	6489	UUCCUCUUAAAGGACACAAGGGACAUCCACAGGAGGGGAUGACUG	0.643	0.718	0.649	0.649	m6A site (High confidence)
21	6554	GGAUGUUAGGUAAAAGGAAAGGACAAAUGGCUGGAGAACUGGUGU	0.612	0.264	0.539	0.565	m6A site (Moderate confidence)
22	6569	GGAAAGGACAAAUGGCUGGAGAACUGGUGUUUCACCCUUCCCUGG	0.603	0.349	0.565	0.575	m6A site (Moderate confidence)
23	6655	CAGGGUCUGGGCUCCCAGGUGGACCAUGAAACCCUGGCCUGACCA	0.59	0.662	0.636	0.612	m6A site (High confidence)
24	6731	GGAGCCCCAGUCCCUGACAAGGACCUAGGACAUUUUUGCUCCUGC	0.611	0.675	0.412	0.534	m6A site (Low confidence)
25	6738	CAGUCCCUGACAAGGACCUAGGACAUUUUUGCUCCUGCCCAGCCU	0.653	0.631	0.474	0.58	m6A site (Moderate confidence)
26	6800	AGCCUUUCAGCUCUGCUGUGUGACUUUGAGGUUGUUGCUCCCCUC	0.489	0.396	0.63	0.541	m6A site (Low confidence)
27	6934	AAGCACAGAAGGGGCAGGAGAGACACUCAGAGGCACUUCCGCUCU	0.531	0.569	0.608	0.564	m6A site (Moderate confidence)
28	6965	GGCACUUCCGCUCUUGCCCAGGACAUUUUCCCAGCCACACCUUUG	0.657	0.731	0.607	0.641	m6A site (High confidence)
29	7187	GGGUGCAGCCAGUCGUGUCCGAACUCUCCAAUGACUAAGCGGGGA	0.634	0.612	0.48	0.571	m6A site (Moderate confidence)
30	7383	AGCGGGGAAGGGGUUCUGAAGAACUCUGGCCAAGAGGACGAGGAU	0.656	0.667	0.387	0.549	m6A site (Low confidence)
31	7696	UAAGAGUCCUGUUGGCUGCAGGACUCAGAGCAUGGACAGGUGGAU	0.668	0.627	0.505	0.601	m6A site (High confidence)
32	7709	GGCUGCAGGACUCAGAGCAUGGACAGGUGGAUAGUAAAUCACCAC	0.713	0.646	0.622	0.673	m6A site (Very high confidence)
33	7739	AUAGUAAAUCACCACCACGGGGACAGCCGUGCCCAGACUGUGCGU	0.575	0.521	0.649	0.602	m6A site (High confidence)
34	7753	CCACGGGGACAGCCGUGCCCAGACUGUGCGUUUGCUUAGCUCGGG	0.668	0.517	0.549	0.613	m6A site (High confidence)
35	7777	UGUGCGUUUGCUUAGCUCGGGGACAGCACUUGGCCCGGGGUCUCC	0.559	0.633	0.536	0.554	m6A site (Low confidence)
36	7829	CUCCCUUCAGAGCAUCUGCCAAACUUCGGGCAUCUACCCUGCAAU	0.452	0.436	0.693	0.548	m6A site (Low confidence)
37	7948	GCUGGAGCUGCUUUGCAGAAUGACUUGGGUCUUGCUGGCCCCUGG	0.537	0.701	0.578	0.561	m6A site (Moderate confidence)
38	8032	UCCCCUUGGUGCCUAACCCAGGACUUUGUCCCCAGAGACCCACUG	0.752	0.786	0.434	0.626	m6A site (High confidence)
39	8141	AGGCCCAAGGAGGUUGUCAGGGACACACAGCAGGGGGAGGCAGCC	0.666	0.798	0.371	0.554	m6A site (Low confidence)
40	8355	UUGGUCCUUACUCCAUGCCAGGACUUGUGCACAUCUUUUUGGAGC	0.656	0.551	0.48	0.581	m6A site (Moderate confidence)
41	8786	GCCCAAGGUCACCACCCUCUGAACUGAGGCGUCCCCAACCCAUGC	0.671	0.616	0.429	0.571	m6A site (Moderate confidence)
42	9344	CGUGACACAAAGAGGGGAGAUGACAGUGGCUGGAGUUGUCAGAGC	0.421	0.368	0.701	0.53	m6A site (Low confidence)
43	9415	AGGCUUGACAGGGCCAAGGGGAACUAUUGUGGAAUGUCUUGGCCU	0.605	0.534	0.61	0.603	m6A site (High confidence)
44	9736	UGACUCACUCGGGAUCCACUGAACUGGGAGGUCUGUGUCUCCUCC	0.644	0.543	0.528	0.593	m6A site (Moderate confidence)
45	9807	CCGCAGGAUCACCCAGCUUGGAACUAGAUACAGAAAUGCUGUUUU	0.549	0.601	0.627	0.583	m6A site (Moderate confidence)

45 m6A residues located across TINCR sequence.

m6A methylation is a characterized process that needs the involvement of m6A reader proteins, including YTHDFs [15]. Given that YTHDF2 participated in the modulation of m6A-dependent RNA degradation [16], we, therefore, assumed that YTHDF2 may be essential for METTL14-mediated TINCR decay and downregulation. By performing RIP using antibody against YTHDF2, we identified the positive binding of TINCR by YTHDF2 antibody in DCM rats (Fig. 6E). Consistently, RNA pulldown assay showed that YTHDF2 was significantly enriched by TINCR (Fig. 6F). Then, we silenced YTHDF2 expression in cardiomyocytes (Fig. 6G). The decay rate of TINCR was significantly slower in shYTHDF2-infected cells (Fig. 6H), indicating that YTHDF2 mediated m6A-RNA decay of TINCR. Altogether, our results revealed that METTL14-mediated m6A modification inhibited TINCR expression via YTHDF2-regulated RNA degradation.

### TINCR promotes pyroptosis and DCM via stabilizing NLRP3 mRNA

**Fig. 7:**
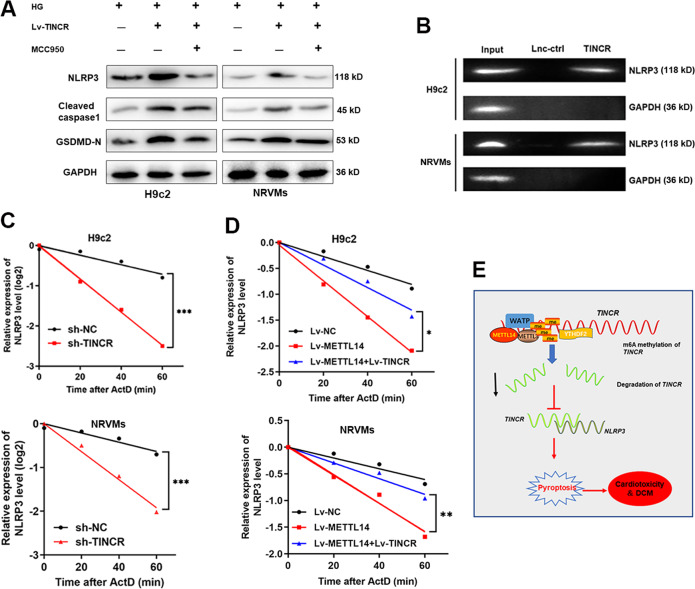
TINCR functions through stabilizing NLRP3 mRNA. **A** Western blot experiment was performed to test the expression of pyroptosis-related proteins in cardiomyocytes treated with Lv-TINCR and MCC950. **B** RNA pulldown using specific TINCR probe revealed a direct interaction between TINCR and NLRP3. **C** Cells were treated with ActD, then existing NLRP3 mRNA was detected at different time point. Silence of TINCR significantly decreased NLRP3 stability, *P* < 0.001. **D** METTL14 suppressed NLRP3 stability, while co-transfection of Lv-TINCR partially reversed this effect, *P < 0.05, **P < 0.01. **E** A scheme of the proposed mechanisms: TINCR was modified by METTL14-mediated m6A methylation, which induced suppression of TINCR in cardiomyocytes. Suppressed TINCR caused decreased stability of NLRP3 and thereby induced its downregulation. Eventually, downregulated NLRP3 inhibited pyroptosis and DCM progression.

Previously, we demonstrated that TINCR regulated cardiomyocytes pyroptosis via stabilizing NRLP3 in DOX-induced cardiotoxicity [13]. To verify whether this regulation mode applies in DCM, we performed gain-and-loss functional assays. As shown, MCC950 remarkedly abrogated the TINCR-regulated pyroptosis in both HG-treated untreated cardiomyocytes (Fig. 7A and Supplementary Fig. S4). To further confirm the direct interaction between TINCR and NLRP3 RNA, we conducted RNA pulldown assay by generating biotinylated oligonucleotides in HG-treated H9c2 and NRVMs cells. As shown, NLRP3 RNA was remarkably enriched by biotinylated TINCR (Fig. 7B). By treatment with actinomycin D (ActD), a well-known inhibitor for RNA transcription, we evaluated the effect of TINCR on downstream mRNA degradation. The results revealed that knockdown of TINCR resulted in accelerated degradation of NLRP3 mRNA in cardiomyocytes (Fig. 7C). More importantly, METTL14 decreased the stability of NLRP3, and overexpression of TINCR reversed this effect (Fig. 7D). Collectively, the above results strongly support that METTL14-mediated m6A induced suppression of TINCR, which further regulates pyroptosis via stabilizing NLRP3 mRNA (Fig. 7E).

## Discussion

It is estimated that DCM occurs in approximately 12% of diabetic patients [17]. Clinical data suggest that DCM is closely associated with Clinical studies that indicate that DCM increases the risk of overt heart failure and induces worse prognosis in diabetic patients [18]. A strategy for prevention and treatment in order to improve the prognosis of DCM has not been established. In this study, we established that METTL14, a well-known m6A writer, inhibited pyroptosis and DCM through downregulation of lncRNA TINCR and NLRP3 expression. Mechanistically, METTL14 mediated m6A modification of TINCR, and thus suppressed TINCR expression and the NLRP3 stability, preventing the occurrence of pyroptosis.

DCM is characterized by structural and functional disorders, including myocardial cell death, myocardial fibroblast activation, left ventricular dysfunction, and metabolic deregulation [19]. Pyroptosis is characterized by programmed cell death inflammation and has been proved crucial for controlling microbial infections [20]. Accumulating evidence suggests that pyroptosis may contribute to a range of diseases, including autoimmune diseases, diabetes mellitus, nervous system-related diseases, and cardiovascular diseases [21–23]. Several reports showed that pyroptosis was closely associated with DCM progression [24]. Moreover, HG treatment could lead to the produce of active oxygen species, resulting in inflammation accompanied with elevated expression of cleaved caspase-1, IL-1β and IL-18 [25]. In addition, the activation of the inflammasome and the release of cytokines can promote the deposition of collagens and fibrotic formation, further exacerbating the severity of DCM [26]. Our study further confirmed that pyroptosis was involved in DCM both in vitro and in vivo. Moreover, this regulation is mainly through the regulation of NLRP3 inflammasome.

The m6A modification is deposited to RNAs by the m6A methyltransferase (writer) complex, a protein complex formed by METTL3/METTL14 heterodimeric catalytic core and a regulatory subunit, WTAP. METTL3 and METTL14 are co-located in nuclear spots and form stable complexes in a 1:1 ratio [27]. METTL14 is a pseudo-methyltransferase that stabilizes METTL3 and recognizes target RNA [28]. Here is emerging evidence to indicate that m6A modification is closely related to the occurrence and progression of CVDs, including cardiac hypertrophy, heart failure, ischemic heart disease, etc. Dorn et al. [10] demonstrated that METTL3-mediated m6A modification is significant for maintaining cardiac homeostasis and normal cardiac function and revealed increased m6A methylation in cardiomyocytes under hypertrophic stimulation. However, whether METTL14 is involved in cardiovascular diseases, such as DCM are largely unknown. Our study confirmed that METTL14 was downregulated in DCM models and remarkably suppressed DCM. More importantly, METTL14 inhibits pyroptosis in a NLRP3-dependent manner, which uncovered a novel functional role of METTL14 in pyroptosis and pyroptosis-induced DCM. Here, we need to point out that the conclusion obtained were based on the rat model induced by streptozotocin injection, which mimics the condition of type 1 diabetes. Whether the regulation of METTL14 in DCM also applies to other DCM models, such as high-fat diet model (T2DM), and what will the functional implications of METTL14 in those modes, need more strong evidence from further studies.

Recent studies have found that, in addition to the roles of m6A modifications in mRNAs, m6A modifications regulate the generation and function of noncoding RNAs, such as lncRNAs. LncRNAs are a class of transcripts more than 200 nucleotides long with no protein-coding function [29]. m6A modifications might modulate the function of lncRNAs by providing a binding site for the m6A reader proteins or by modulating the structure of the local RNA to induce RNA-binding protein entry. Previous study showed that knocking down METTL3 reduced the level of m6A modifications on specific transcripts such that the lncRNA X chromosome was inactivated [30]. Gone et al. demonstrated that METTL14 mediated m6A modification suppressed LncRNA ZFAS1/ RAB22A expression and provided novel therapeutic targets for atherosclerosis [31]. Many lncRNAs show temporal differential expression, and display genic distribution in the genome. Interestingly, temporal-specific m6A-methylation with consensus m6A motif GGACU was reported in the last exon in most lncRNAs, indicating the potential way of interaction between m6A-methylation and lncRNA expression [32]. Our former study revealed the essential role of lncRNA TINCR in pyroptosis and myocardial damage. With the consistent functions between METTL14 and TINCR, we investigated the potential regulatory mode of METTL14 and TINCR. By predicting with an online software, we uncovered several potential binding sites of m6A. RIP assay further confirmed the direct interaction.

The current hypothesis suggests that for m6A modification to exert its biological functions, it must first be recognized by m6A reader proteins. Human YTH domain family proteins include three members, YTHDF1-3, each of which comprises a highly conserved single-stranded RNA-binding domain located at the carboxy terminus (the YTH domain) and a less conserved amino-terminal region [33]. The fates of m6A modified mRNAs are dependent on m6A selective binding proteins [34]. YTHDF2 is the first identified and well-studied functional m6A-binding protein that mainly regulates the stability of mRNA [35]. YTHDF2 recognizes and binds to m6A sites in 3’UTR of mRNA through its C-terminal YTH domain to accelerate the degradation of target mRNAs [36]. As METTL14 decreased the expression of TINCR, we supposed the m6A reader could be YTHDF2 and finally, this hypothesis was confirmed, which further strengthen our previous report.

The regulation of NLRP3-based pyroptosis by TINCR has been well defined in DOX-induced myocardial damage in our previous study. Importantly, the regulation mode also applies to DCM. This commonly used pathway in pyroptosis suggests a tight correlation between TINCR-NLRP3 pathway and pyroptosis regardless of the causes of pyroptosis. However, the detailed mechanism by which DCM/HG-induced higher expression of TINCR is not well known, which is the limitation of this study. We will follow this study and continue exploring the underlying pathways. In addition, the expression of METTL14 in a healthy population and in different organ of diabetic patients with/without organ damage could be an interesting topic in the area of diabetics. We will focus on this issue and keep exploring the regulatory mechanism of METTL14-mediated pyroptosis in diabetic patients to identify its potential clinical value for predicting organ damage.

In conclusion, we demonstrated that METTL14 suppresses pyroptosis and DCM progression via m6A methylation of TINCR mRNA in an NLRP3-dependent manner. Our study not only help better understand the regulatory mechanism of METTL14-mediated m6A modification in myocardial damage but is also useful for finding promising drug targets and developing novel therapeutic strategies to overcome DCM.

## Supplementary information

Supplementary Figure legends

Supplementary Figure S1

Supplementary Figure S2

Supplementary Figure S3

Supplementary Figure S4

checklist

## Data Availability

The analyzed data sets generated during the study are available from the corresponding author on reasonable request.
