# Correction: METTL14 suppresses pyroptosis and diabetic cardiomyopathy by downregulating TINCR lncRNA

**DOI:** 10.1038/s41419-025-07680-3

**Published:** 2025-05-26

**Authors:** Liping Meng, Hui Lin, Xingxiao Huang, Jingfan Weng, Fang Peng, Shengjie Wu

**Affiliations:** 1 Department of Cardiology, Shaoxing People’s Hospital(Shaoxing Hospital, Zhejiang University School of Medicine), Shaoxing, 312000 Zhejiang China; 2 Department of Cardiology, the First Affiliated Hospital of Wenzhou Medical University, Wenzhou, China

Correction to: *Cell Death and Disease* 10.1038/s41419-021-04484-z, published online 10 January 2022

During our subsequent research, we noticed that the images of the Calcein-AM staining of H9C2 in Fig. 1B were placed incorrectly. We have revised the errors and shown the corrected images in the corrected Fig. 1B. All of our authors confirmed that this revision will not change our results and conclusions. The authors would like to apologize for any inconvenience caused.

**Original Figure 1** 
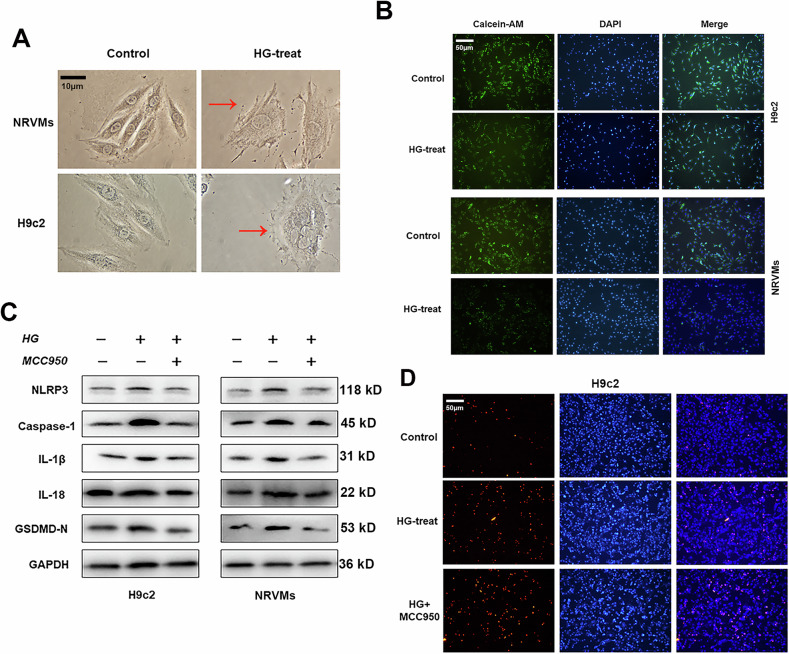


**Corrected Figure 1** 
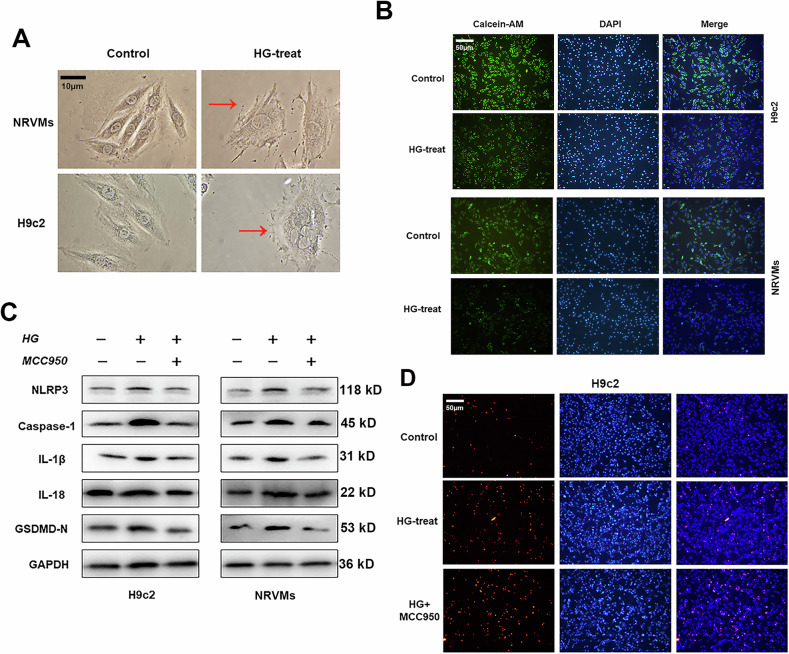


The original article has been corrected.

