# Supplementary material for: METTL14 suppresses pyroptosis and diabetic cardiomyopathy by downregulating TINCR lncRNA
Source: Cell Death Dis. 2022 Jan 10;13(1):38. doi: 10.1038/s41419-021-04484-z (PMC8748685; doi:10.1038/s41419-021-04484-z)
Supplement: Supplementary file 1 — Supplementary Figure legends [file 41419_2021_4484_MOESM1_ESM.docx]

**Supplementary Figure S1 The effects of NLRP3 inhibitor MCC950 and hyperosmotic pressure on DCM.**

**A.** Expression of pyroptosis-related proteins was detected in H9c2 and NRVMs cells upon treatment with 25 mmol/L glucose for 24 h. **B.** Quantification of damaged cells upon HG treatment, ^***^P<0.001. **C**. Quantification of expression of indicated proteins normalized to GAPDH, ^*^P<0.05, ^**^P<0.01, ^***^P<0.001. **D**. The proportion of apoptotic cells in respective groups, MCC950 was used at the concentration of 7.0 nM. **E**. IHC analysis was performed to detect the expression of NLRP3 in rats treated with MCC950 (12 mg/kg). Results showed that MCC950 significantly suppressed NLRP3 expression levels. **F**. Cardiac function analysis of rats treated with MCC950, and the results showed that MCC950 treatment showed no effect. **G.** Blood inflammatory factors, such as CK-MB, LDH and AST were not influenced by MCC950 treatment. **H.** Western blot analysis showed that pyroptosis-related proteins were not dysregulated under different hyperosmotic conditions.

**Supplementary Figure S2** **Expression of METTL3 and METTL14 in hyperosmotic and HG conditions.**

**A**. Western blot analysis of METTL14 expression in H9c2 cells treated with D-sorbitol at the concentration of 200, 400 and 800 mM to mimic hyperosmotic conditions. Results showed that METTL14 was not significantly dysregulated by hyperosmotic conditions. **B**. Western blot analysis of METTL3 level in cardiomyocytes and showed that METTL3 was not regulated by HG treatment. **C**. IHC analysis of METTL3 protein in rats with DCM, and the data indicated that METTL3 level was not altered in DCM rats compared to controlled ones.

**Supplementary Figure S3A-C** Silence of TINCR reversed sh-METTL14-induced pyroptosis in normal rats, including the pyroptosis-related protein levels (A), cardiac functions (B) and ultrastructure damage.

**Supplementary Figure S4** MCC950 treatment reversed the TINCR-induced upregulation of proteins involved in NLRP3 pathway in cardiomyocytes without HG treatment.
