# Supplementary figures and images for: METTL14 suppresses pyroptosis and diabetic cardiomyopathy by downregulating TINCR lncRNA
Source: Cell Death Dis. 2022 Jan 10;13(1):38. doi: 10.1038/s41419-021-04484-z (PMC8748685; doi:10.1038/s41419-021-04484-z)

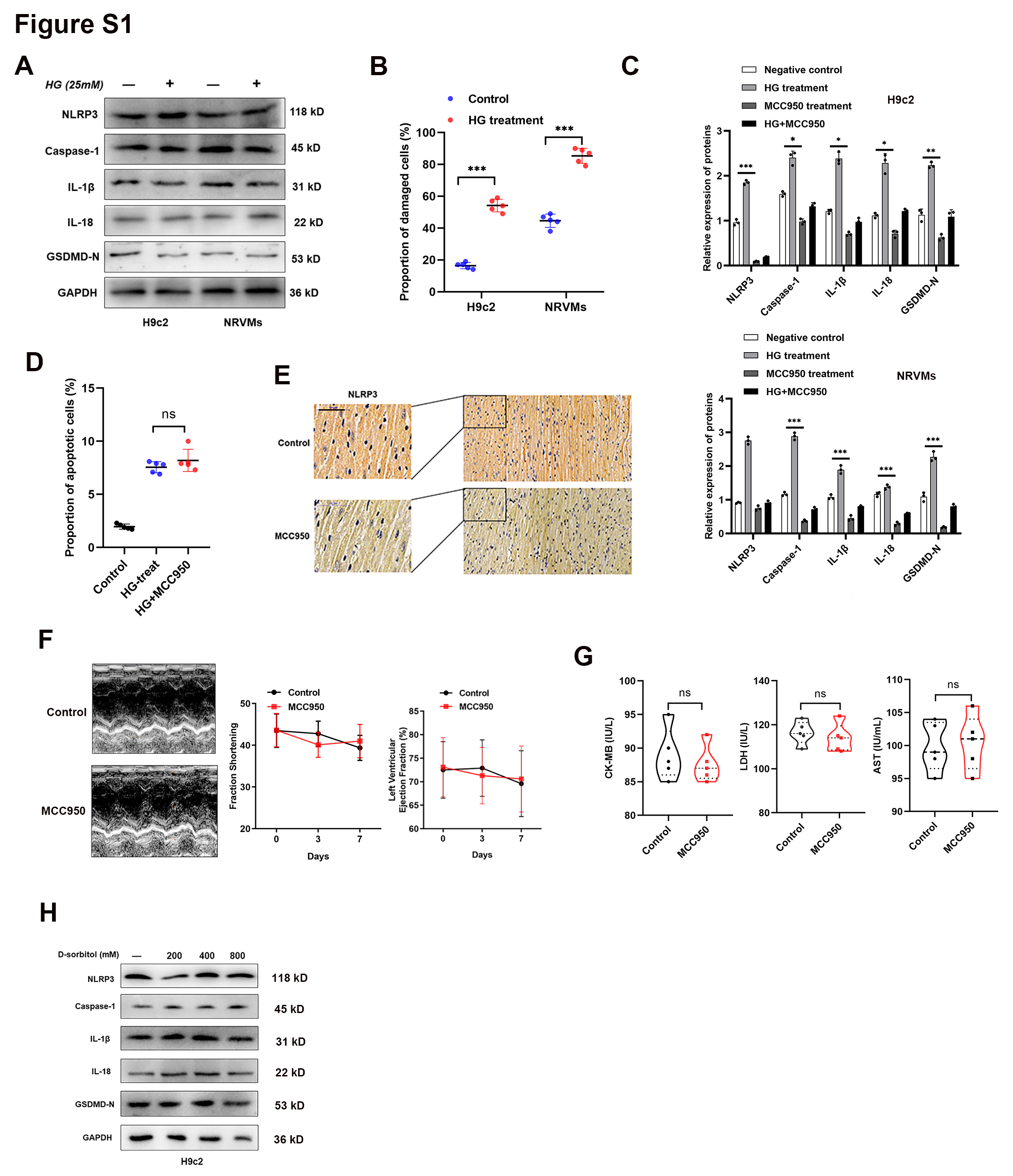

Supplement: Supplementary file 2 — Supplementary Figure S1 [file 41419_2021_4484_MOESM2_ESM.tif]

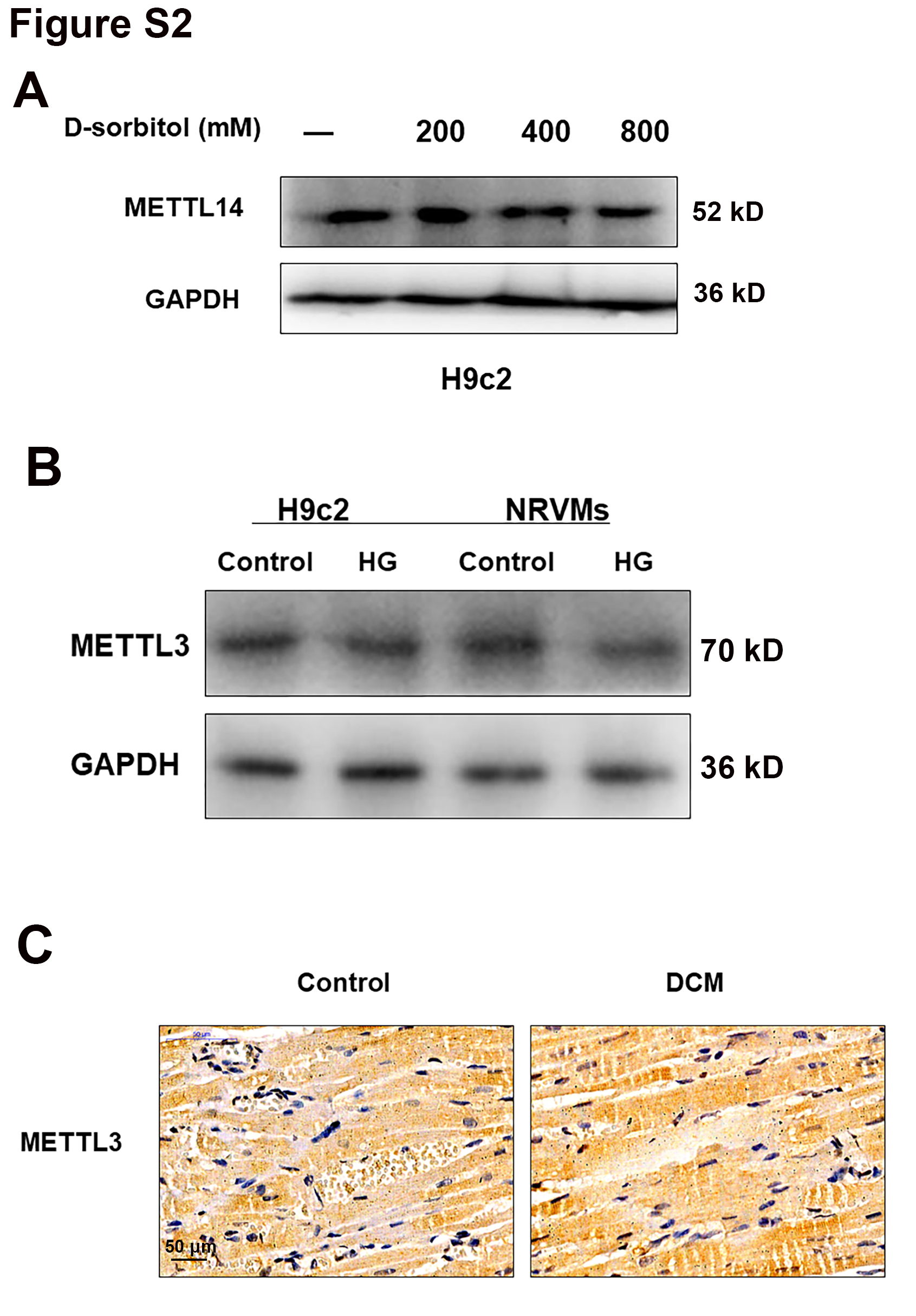

Supplement: Supplementary file 3 — Supplementary Figure S2 [file 41419_2021_4484_MOESM3_ESM.tif]

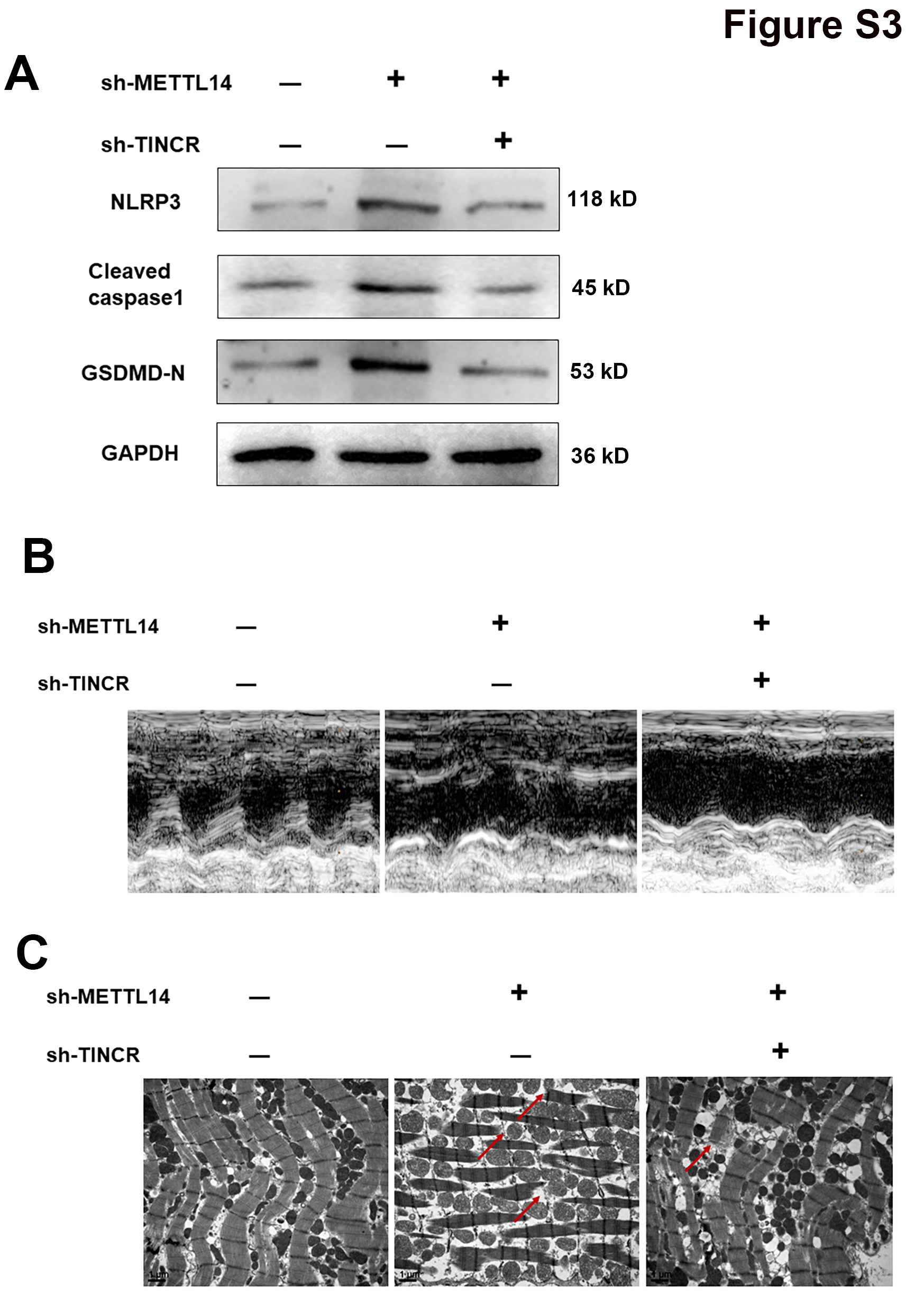

Supplement: Supplementary file 4 — Supplementary Figure S3 [file 41419_2021_4484_MOESM4_ESM.tif]

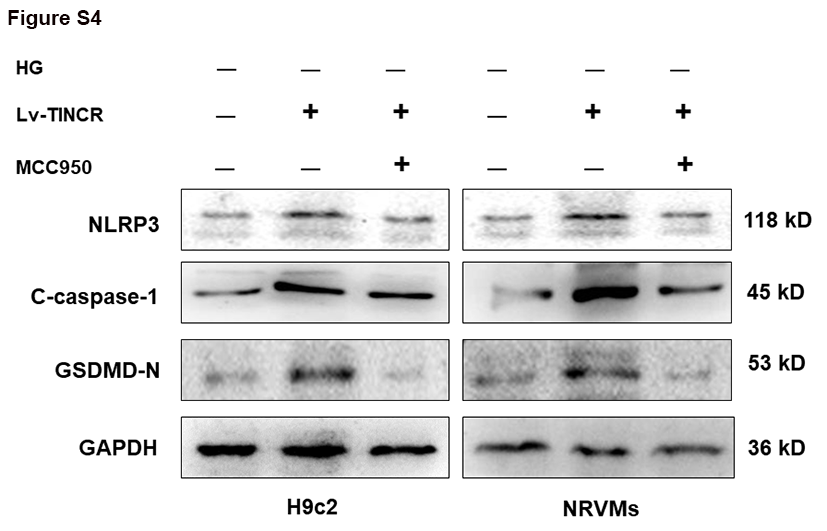

Supplement: Supplementary file 5 — Supplementary Figure S4 [file 41419_2021_4484_MOESM5_ESM.tif]
